# The Association between Patient-Reported Pain and Doctors' Language Proficiency in Clinical Practice

**DOI:** 10.1155/2015/263904

**Published:** 2015-09-21

**Authors:** Marianne Mustajoki, Tom Forsén, Timo Kauppila

**Affiliations:** ^1^Department of General Practice and Primary Health Care, University of Helsinki, Kiskontie 23 B, 00280 Helsinki, Finland; ^2^Department of General Practice and Primary Health Care, University of Helsinki, PB 20 (Tukholmankatu 8 B), 00014 Helsinki, Finland

## Abstract

Patients' limited literacy and language fluency of different kinds cause them problems in navigating the medical interview. However, it is not known how physicians' native language skills affect the reported intensity of pain among Finnish emergency patients. Data were collected with two consecutive questionnaires in 16 healthcare centres and outpatient departments along the Finnish coast. Swedish and Finnish speaking 18–65-year-old emergency patients were eligible for this study. Our patients were predominantly Finnish speakers. Patient-rated poor language skills in Finnish among the physicians in ED setting increased statistically significantly pain reported by the Finnish speaking patients and their dissatisfaction with the health service. These patients were also less motivated to adhere to the instructions given by their physician. Patients speaking various languages reported less degree of pain. Foreign physicians' poor language proficiency in Finnish was expected to explain only some of the patients' pain experience. Physicians' good native language skills may help to reduce pain experience. Despite concordant language communication, other unknown barriers in the interaction might reduce the magnitude of pain reported.

## 1. Introduction

Healthcare is ideally delivered in language concordance which means that both the patient and the physician speak the same preferred language. Mutual language and understanding are critical in generating good functional relationships between health staff and patients. Language barriers cause communication difficulties which may hamper the treatment of a disease [1]. Patients with difficulties to express themselves in a nonnative language are less adherent to health instructions and report significantly decreased patient satisfaction [2–5]. Furthermore, communication problems with the patients affect also the physicians and impede their decision making and adequate medical treatment. On the other hand, limited literacy and insufficient language fluency cause the patients problems in navigating the medical interview. Diagnosing, for example, acute chest pain patients in emergency departments (ED) may be hampered because of language barriers [6]. In line with this, language barriers have been associated with a higher rate of resource utilization for the diagnostic process and increased ED visit times [7]. Yet common cultural and lingual conditions do not necessarily prevent the patients and physicians from miscommunication, thus compromising mutual understanding. Patients' limited comprehension of their disease can also undermine effective communication and distract physicians from investigating symptoms. The patients and physicians might have different explanations for diseases which are reflected in the clinical information gained in the medical interview. Patients suffering from chronic diseases may also be exposed to cognitive problems affecting their ability to express reliably vital symptoms such as pain [8, 9].

Measuring the pain intensity in a clinical situation is challenging and as a rule healthcare personnel underestimate the severity of pain [10, 11]. In line with this, a paired survey demonstrated that socially discordant physician-patient interaction resulted in the physicians' overestimation of patients' confidence and trust but underestimation of their pain. The physicians' inability to effectively communicate with their patients may lead to frustration, which in turn leads to the patients' increasing concern of not being heard [12]. On the other hand, the physicians' ability to assess pain severity has been reported not to differ for Hispanic and non-Hispanic white patients in an ED in the USA, suggesting that there may be other explanations for observed differences in analgesic practice than ethnicity-based misinterpretation of the patients' pain intensity [13]. However, the physicians' language skills might affect the estimation of pain, resulting in poorer diagnostic confidence and increased need of ancillary tests [14]. Foreign physicians are not automatically able to fluently communicate in the patients' native language. The amount of mainly native Russian and Estonian speaking physicians has increased during recent years in Finland and they are likely to be overrepresented in EDs. A majority of them report very good communication skills in Finnish, but no data are available about their proficiency in Finland's second national language Swedish [15]. Thus this study about patients' experience concerning the physicians' communication skills in an ED setting and the effects on estimation of pain severity is a relevant issue in Finland.

By tradition Finland is an ethnically broadly homologous but bilingual country where patients have the right to get health service either in Finnish or in Swedish. The great amount of language contacts had exposed the minority of Swedish speakers to increasing Finnicization (“language transition”) [16]. This linguistic instability typical for Finland means that the linguistic exchange in public services generally proceeds in Finnish and not in Swedish. Thus we have good opportunities to study how exclusively linguistic factors impact on the communication in EDs. As the Swedish speaking minority in Finland lives along the south and southwest coasts intermingled with the Finnish speaking majority this exceptional setting is ideal for testing the importance of linguistic factors in pain measuring. More important for the present study is that, unlike the conditions for minorities in general, these two language groups studied here—the Swedish speakers or Finnish speakers Finns—are quite similar in most aspects, including socioeconomic status, education, religion, and access to health services [17, 18]. According to this we were able to specifically study how the patients' and physicians' language skills affect the intensity of patient-reported pain.

## 2. Materials and Methods

Our study was approved by the Ethics Committee of the Hospital District of Helsinki and Uusimaa (Reference number 5/13/03/00/2008). Data were collected in 2008-2009 in 15 healthcare centres and outpatient departments along the south coast and in one healthcare centre in South Ostrobothnia. Only Swedish and Finnish speaking 18–65-year-old emergency patients were eligible for this study. Patients with major mental disturbances as well as life threatening symptoms were excluded by the personnel.

Everyone visiting healthcare in Finland is registered electronically. The personal data in the registration for population are automatically transferred into everyone's electronic case record. These data contain also information about the patients' native language. The arriving emergency patient was informed by a receptionist or a nurse about the possibility to voluntarily participate in the study. Interested and applicable patients were provided with language concordant information about the study and an agreement-form plus a questionnaire. All corresponding data-collection material was also accessible on a table in the waiting-room for entering patients in order to facilitate participation by oneself. The patients completed the questionnaire before the physician's appointment and were advised to drop the sealed questionnaire into a locked box in the waiting-room. The questionnaire included 43 closed questions, of which 15 were standardised questions about socioeconomic and health conditions used in periodical population surveys in Finland; 23 questions about native and nonnative language proficiency, the relatives' native language, language spoken at home and stated in registration for population, Finnish or Swedish schooling, and preferred communication language with the general practitioner (GP); and 5 questions about the frequency and quality of health centre visits and the reason for the visit.

Two weeks after the emergency visit, the patients who agreed to a follow-up were sent a second questionnaire including detailed filling instructions by mail. The language in the second questionnaire was specified according to the patient-reported native language during the visit. This structured questionnaire included 30 closed questions about the GP's language proficiency in the patient's native language and patient-preferred communication language, the patient's feeling of confidence and satisfaction on a 1–5 graded scale, pain experience on standardized VAS scale, frequency of laboratory tests and X-rays during the visit, medication prescriptions, pain medication, written and verbal instructions in the patient-preferred language, and length of sick leave. To quantify the language discordance between the patients and the GPs, the patients were asked to assess the GP's language proficiency in Finnish or Swedish according to a numerical, well-established Finnish school grade of 4–10. The patients' numeric ratings of the GP's language proficiency in the patient-preferred language were pooled into good, average, and poor by means of a data reduction technique for statistical analysis [19]. A measure of the patients' language proficiency was obtained by asking them to assess their ability to communicate in their second nonnative language.

Data were statistically analysed with SPSS system. Correlations between variables were calculated using linear regression. Statistical significance was set at *p* < 0.05. Adjustment was used for age, income, education, and gender.

## 3. Results

875 patients in total filled in the first questionnaire during their emergency visit on the healthcare centres. 53% of them (*n* = 466), predominantly Finnish speaking female patients, replied to the second questionnaire. All respondents did not reply on every question in the questionnaire. Tables include only individuals with available data.

The characteristics of the respondents reveal that the Swedish speaking respondents were on average ten years older than the Finnish speaking respondents (Table 1).

The statistical analyses were performed according to the patient-preferred language stated in the questionnaire by the respondents. The Swedish speaking patients were less likely to estimate the GP's language proficiency in their native language since they generally communicated in Finnish. 55% of the patients reported that the GP's native language was Finnish, 10% Swedish, and 35% another language rather than Finnish or Swedish.

Comparison between Swedish speakers (*n* = 24) and Finnish speakers (*n* = 407) revealed that the Swedish speakers were significantly less confident about the GP's professional qualification (*p* < 0.001) and the care quality (*p* < 0.001) during the visit. They also reported significantly less motivation to adhere to the GP's instructions (*p* < 0.001) (Table 2). The Swedish speakers reported less pain when they were treated by a Swedish speaking GP than did the Finnish speakers although the difference did not reach the level of significance (Table 3).

The Finnish speakers reported significantly less unspecified pain when the GP's language proficiency in Finnish was good. On the contrary, the GP's poor language skills in Finnish increased significantly the degree of patient-reported pain in all diseases, except in musculoskeletal diseases (*p* < 0.01) (Table 4). The patients with good proficiency in a second nonnative language reported less unspecified pain compared to patients who knew only one language (Table 5). One-third of all patients were prescribed analgesics, but the small sample size made analysis of the significance between language groups impossible.

The GP's language proficiency in patients' native language, in both Finnish and Swedish, influenced the patients' experiences of the emergency visit. Deficient language competence among the GPs tended to increase both Finnish speakers' and Swedish speakers' dissatisfaction with the emergency visit and their insecurity, uncertainty, and fear during the visit. Furthermore, those patients were significantly less motivated to adhere to the instructions (*p* < 0.001) given by the GP (Table 6). One-third of the patients had undergone laboratory tests and 12% an X-ray during the visit.

## 4. Discussion

Our results suggest that the patients report a lower level of pain if they have estimated the GP's language skills highly in the patient-preferred language. This result is in line with previous findings among Spanish speaking cancer patients in the USA [20]. We also found that the patients' ability to speak an additional nonnative language was associated with a lower degree of reported pain. Patients with good nonnative language proficiency seemed to have generally advantageous communication conditions. These findings were independent of the GP's patient-rated language proficiency.

Patients' higher level of self-efficacy for pain communication has been reported to be associated with significantly lower levels of pain, physical and psychological disability, and pain catastrophizing and with lower levels of partner negative affect [21]. In the light of these findings, our results regarding the reasons for more intensive patient-reported pain are not surprising, although all pain decreasing factors are not known. Our results can, however, also indicate that the patients' estimation of the GP's language skills mirrors a plausible social discordance between the patients and GPs. We suppose, furthermore, that the patients' impression of the GP's personality is likely to influence patients' estimation of the GP's language proficiency.

The perception of the interaction and different ethnicity between the patient and the GP tends to affect the GP's perception of patients' pain [22]. Unfortunately, we did not collect any data about the GPs' nationalities from the healthcare centres, and so we do not know how many patients visited a nonnative (e.g., Swedish or Finnish is not the mother tongue) GP. From the patients' spontaneous remarks in the questionnaires we could, however, conclude that some foreign GPs were on duty during the study time indicating that lingual and cultural disparities might cause some patients' communication problems including uncertainty and fear. This topic would be exceptionally difficult to study with minorities in several other communities. However, the equal socioeconomic conditions among the broadly homologous but bilingual population living along the south coast and in South Ostrobothnia region provide an ideal test group for our study [16–18].

Our study revealed that, despite concordant language communication, other barriers had impact on reported pain intensity. As noted, low literate patients have less capability to spontaneously relate their symptoms in a structured and precise way. Our respondents had between 13.0 and 14.5 years of education and thus low literacy seems unlikely to cause any major problems in the medical interview. The GPs' preference to use difficult words, mainly Latin, when explaining the origin of disease might, however, also cause the patients uncertainty about the severity of their symptoms.

Greater depth of patient-physician relationship in primary care has been suggested to increase the GPs' detection of patients' emotional distress [23]. Altogether 41% of our respondents reported having earlier visited an assigned GP. One emergency patient out of five had the ED visit made to a previously assigned Finnish speaking GP. Our study, however, could not demonstrate less patient-reported pain among those patients who met their assigned GP during their emergency visit.

Although pain is an important symptom in most diseases, the emergency situation might prevent especially stressed patients from being active in the medical interview. Our findings can also indicate that increased patient-reported pain reflects GPs' emphasising laboratory tests or X-rays instead of asking the patients what is wrong with them. Compared to Finnish data from 1998 our study could not demonstrate the GPs compensating poor communication by a need of more tests to narrow the diagnosis [24].

The patients reported more pain when the GP's language proficiency was poor in all other reasons for the visit other than musculoskeletal diseases. Obesity (body mass index, BMI, > 30 kg/m^2^) has been noted to increase the patient-reported pain compared to normal-weight and underweight patients [25]. Our respondents reported average BMI < 30 kg/m^2^ indicating that obesity was not the exclusive explanation for pain. Furthermore, pain is the most prominent symptom in musculoskeletal diseases and therefore is likely to be noted by the GP in the medical interview, but we assume that urological, gynaecological, obstetrical, and abdominal symptoms including pain might embarrass many patients minimizing their information especially if the communication with the GPs is poor. Unquoted and unexplained pain can increase the patients' worries for the situation which in turn intensify the symptoms. Being disbelieved by healthcare providers or reassured that nothing is physically wrong has also been noted to worsen symptoms [26]. Many symptoms are, furthermore, difficult for patients to identify and explain without GPs' verbal navigation. Any significant differences in GPs' prescription of analgesics and the patient-preferred language were not possible to demonstrate in this study as the Swedish speaking patients were few. However, based on earlier minority studies, we assume that a larger sample size could reveal putative significance. By paying more attention to the GPs' language education, language barriers are possible to reduce in healthcare service.

## 5. Conclusions

The GPs' deficient native language proficiency affects negatively patients' experience of the emergency visit which might affect also pain communication. Our study could thus verify that although language concordant communication is a prerequisite for mutual understanding, pain revealing requires additionally good language skills.

Previous studies have consistently confirmed that language minority patients are in general at risk for communication problems in healthcare mainly caused by their unfavourable socioeconomic conditions as well as cultural and lingual disparities. We could, however, demonstrate that language discordance alone in a sociocultural homogenous population is sufficient to increase both minority and majority patients' insecurity and fear and can also intensify patient-reported pain.

## Study Limitations and Strengths

The strengths of this study were that the respondents represented typical acutely ill native patients making out-of-hour visits to healthcare centres in Finland. The coastal region of Finland is an ethnically and culturally relatively homogenous but bilingual part of the country providing exceptionally favourable opportunities to study how linguistic factors impact pain measuring in EDs.

A considerable proportion of our Swedish speaking minority patients reported impediments during the medical interview performed in a nonpreferred language. Our sample was, however, too small to reach the level of significance due to minority patients' reported pain. Further studies are required to reveal how often pain other than that caused by musculoskeletal diseases remains unobserved by the GPs.

## Figures and Tables

**Table 1:** Characteristics of the respondents.

	Finnish speakers (*n* = 383)	Swedish speakers (*n* = 79)
Gender		
M/F (*n*)	80/303	25/54
M/F (%)	21/79	32/68
Age, yrs (mean), M/F	46.0/43.0	56.0/49.8
Annual income (%)		
0–20 000	44.1	54.2
20 001–30 000	32.7	30.6
>30 000	23.1	15.3
Education, yrs (mean), M/F	13.4/14.2	12.2/13.2
BMI (mean kg/m^2^), M/F	28.0/25.9	2.6/26.6

**Table 2:** Correlations between the GP's patient-reported language proficiency (1 = poor, 2 = average, and 3 = good) and the patients' experience of the visit (scale 1–5^*∗*^).

	Mean ± SD (*n*)^*∗∗*^	Total	*p* value
Very secure	2.8 ± 0.5 (155)	2.6 ± 0.6 (432)	<0.001
Secure	2.6 ± 0.6 (172)		
Neither secure nor insecure	2.4 ± 0.7 (81)		
Insecure	2.4 ± 0.7 (21)		
Very insecure	1.5 ± 1.0 (4)		

Very fearless	2.6 ± 0.6 (259)	2.6 ± 0.6 (420)	0.02
Fearless	2.5 ± 0.6 (87)		
Neither afraid nor fearless	2.5 ± 0.7 (45)		
Afraid	2.3 ± 0.8 (16)		
Very afraid	2.6 ± 0.8 (14)		

Great confidence in the GP's skills	2.8 ± 0.5 (122)	2.6 ± 0.6 (433)	<0.001
Confidence	2.7 ± 0.5 (176)		
Neither confident nor uncertain	2.4 ± 0.7 (92)		
Uncertain	2.7 ± 0.7 (30)		
Weak confidence	1.7 ± 0.9 (13)		

Very satisfied with the service	2.8 ± 0.5 (151)	2.6 ± 0.6 (435)	<0.001
Satisfied	2.6 ± 0.6 (135)		
Neither satisfied nor dissatisfied	2.5 ± 0.7 (88)		
Dissatisfied	2.4 ± 0.8 (38)		
Very dissatisfied	2.1 ± 0.8 (23)		

Very motivated to follow the GP's instructions	2.7 ± 0.5 (242)	2.6 ± 0.6 (434)	<0.001
Motivated	2.5 ± 0.7 (129)		
Neither motivated nor unmotivated	2.5 ± 0.7 (43)		
Unmotivated	2.4 ± 0.8 (14)		
Very unmotivated	1.8 ± 1.0 (6)		

^*∗*^1–5 graded scale: 1 = the most negative experience, 5 = the most positive experience.

^*∗∗*^Adjusted for age, gender, income, education, and native language.

**Table 3:** Correlations between the GP's patient-reported proficiency in Swedish and Finnish^*∗*^ and the patients' experience of pain^*∗∗*^.

	Poor	Average	Good	*p* value
The GP's language proficiency in				
Swedish % (*n*)	76.9 (13)	19.0 (12)	60.3 (38)	
Finnish % (*n*)	5.1 (19)	26.1 (98)	68.8 (258)	
Both Swedish and Finnish % (*n*)	7.3 (32)	24.8 (109)	67.6 (296)	
The patients' pain experience mean ± SD (*n*)				
GP's proficiency in Swedish	3.7 ± 2.43 (12)	2.7 ± 2.0 (11)	2.8 ± 1.9 (33)	Ns
GP's proficiency in Finnish	4.4 ± 1.7 (18)	3.7 ± 1.9 (91)	3.3 ± 2.1 (252)	0.005
Proficiency in Swedish and Finnish	4.1 ± 2.0 (30)	3.7 ± 1.9 (103)	3.3 ± 2.1 (285)	0.007

^*∗*^Language proficiency scale: 1 = poor, 2 = average, and 3 = good.

^*∗∗*^Adjusted for age, gender, income, education, and native language.

**Table 4:** Correlations between the GP's patient-reported language proficiency^*∗*^ in Swedish and Finnish and pain experience (pain scale VAS 0–10) related to the reason for emergency visit^*∗∗*^.

	Pain experience, mean ± SD (*n*) when the GP's language proficiency was as follows			*p* value
	Poor	Average	Good	
Reason for visit				
Musculoskeletal problems	5.1 ± 1.5 (6)	4.7 ± 1.0 (27)	4.3 ± 1.8 (77)	0.2
Other health problems	3.8 ± 2.0 (23)	3.3 ± 2.0 (75)	2.9 ± 2.1 (202)	0.01
All problems	4.1 ± 2.0 (30)	3.6 ± 1.9 (102)	3.3 ± 2.1 (285)	0.007

^*∗*^Language proficiency scale: 1 = poor, 2 = average, and 3 = good.

^*∗∗*^Adjusted for age, gender, income, education, and native language.

**Table 5:** Correlation between the patient's pain experience and their language proficiency in a nonnative language^*∗*^.

	VAS pain scale 0–10	Mean ± SD (*n*)	*p* value
The patients' nonnative language proficiency, 1–4 graded scale (1 = none/poor, 4 = fluent)	None or very poor proficiency	3.6 ± 1.9 (78)	
	Speaking satisfactory well	3.5 ± 2.0 (132)	
	Speaking well	3.2 ± 2.1 (121)	
	Fluent proficiency	3.0 ± 2.0 (66)	
	Total	3.4 ± 2.0 (396)	0.02

^*∗*^Adjusted for age, gender, income, education, and native language.

**Table 6:** Correlations between the patients' native language and their experience during the visit (1–5 graded scale^*∗*^).

	Swedish speakers	Finnish speakers	*p* value
	Mean ± SD (*n*)^*∗∗*^	Mean ± SD (*n*)^*∗∗*^	
Sense of security/insecurity	4.23 ± 0.9 (77)	4.0 ± 0.9 (379)	0.99
Trust/fear	1.5 ± 1.0 (71)	1.7 ± 1.0 (372)	0.7
Confidence in/uncertainty of the GP's skills	3.8 ± 1.0 (76)	3.8 ± 1.0 (381)	0.1
Motivated/unmotivated to follow the GP's instructions	4.2 ± 1.0 (76)	4.5 ± 0.9 (381)	0.005
Satisfied/dissatisfied with the service	3.9 ± 1.4 (77)	3.8 ± 1.2 (382)	0.27

^*∗*^1–5 graded scale: 1 = the most negative experience, 5 = the most positive experience.

^*∗∗*^Adjusted for age, gender, income, and education.
